# Metabolic Dysfunction-Associated Steatotic Liver Disease in Patients with Inflammatory Bowel Diseases: A Pilot Study

**DOI:** 10.3390/life14101226

**Published:** 2024-09-25

**Authors:** Ludovico Abenavoli, Rocco Spagnuolo, Giuseppe Guido Maria Scarlata, Maria Luisa Gambardella, Luigi Boccuto, Nahum Méndez-Sánchez, Francesco Luzza

**Affiliations:** 1 Department of Health Sciences, University “Magna Græcia”, Viale Europa, 88100 Catanzaro, Italy; spagnuolo@unicz.it (R.S.); giuseppeguidomaria.scarlata@unicz.it (G.G.M.S.); marialuisa.gambardella@studenti.unicz.it (M.L.G.); luzza@unicz.it (F.L.); 2 Healthcare Genetics and Genomics Doctoral Program, School of Nursing, College of Behavioral, Social and Health Sciences, Clemson University, Clemson, SC 29634, USA; lboccut@clemson.edu; 3 Faculty of Medicine, National Autonomous University of Mexico, Mexico City 04510, Mexico; nmendez@medicasur.org.mx

**Keywords:** Crohn’s disease, ulcerative colitis, liver ultrasound, metabolic dysfunction-associated fatty liver disease

## Abstract

Background: Inflammatory bowel disease (IBD) is characterized by persistent inflammation and is often associated with metabolic dysfunction-associated steatotic liver disease (MASLD). IBD patients are at risk of developing MASLD due to shared risk factors such as gut dysbiosis and systemic inflammation. The new MASLD nomenclature emphasizes the link between liver steatosis and cardiometabolic comorbidities. However, the prevalence of MASLD in IBD patients remains poorly explored. The main aim of this cross-sectional study is to assess the prevalence of ultrasound (US) and the clinical features of MASLD in patients with IBDs. Materials and Methods: We conducted a retrospective study enrolling 272 Italian IBD patients attending Renato Dulbecco Teaching Hospital in a period between 1 January 2021 and 31 December 2023. MASLD was diagnosed based on the presence of liver steatosis with cardiometabolic risk factors, using established guidelines. Demographic, clinical, and laboratory data were collected and analyzed. Statistical significance was determined at a *p*-value < 0.05. Results: Of the 272 IBD patients, 6% had non-alcoholic fatty liver disease (NAFLD), while 18% had MASLD. Patients with IBD-MASLD were significantly older, had higher body mass index, waist circumference, and triglyceride levels, and were more likely to have type 2 diabetes mellitus and hypertension compared to those with IBD-NAFLD. IBD-MASLD patients also showed higher disease activity scores and required more frequent surgical interventions. Bivariate logistic regression revealed triglyceride levels as a significant predictor of MASLD in IBD patients. Conclusions: MASLD is more prevalent in IBD patients, highlighting the importance of early detection of liver steatosis in this at-risk population. The association between MASLD and cardiometabolic risk factors underscores the need for a multidisciplinary approach to manage these patients effectively. Further studies in larger cohorts are necessary to confirm these findings and explore the pathophysiological mechanisms involved.

## 1. Introduction

Inflammatory bowel disease (IBD) includes two different conditions, ulcerative colitis (UC) and Crohn’s disease (CD), characterized by persistent inflammation and subsequent gastrointestinal tract damage [1,2]. IBDs pose a significant public health challenge due to their global prevalence. In Europe, the prevalence of CD ranges from 1.5 to 213 cases per 100,000 people, while UC occurs at a rate of 2.4 to 294 cases per 100,000 individuals [3]. The typical symptoms of IBD, such as abdominal pain, bloating, and altered bowel habits, often overlap with irritable bowel syndrome (IBS), which, despite being a distinct pathological entity, requires a differential diagnosis. Indeed, a recent meta-analysis based on 27 studies showed that IBS symptoms were present in 32% of cases of IBD patients in remission [4]. Additionally, IBS and IBD predominantly affect young adults and have a higher prevalence in females, indicating a demographic similarity [5]. In an observational study, the Authors found a significantly higher incidence of IBD among patients with IBS. The incidence rate of IBD was 8.6 times higher (*p* < 0.0001) in individuals with IBS, at 238.1 per 100,000 person-years, compared to 27.8 per 100,000 person-years in the general population. At the same time, in IBS cases, the risk of developing IBD was higher, with a 15-fold increase compared to subjects without IBS [6]. Furthermore, several additional manifestations beyond the intestine may affect IBD patients, including joint pain, skin conditions, oral ulcers, and non-alcoholic fatty liver disease (NAFLD) [7]. This condition results from the triglyceride accumulation in over 5% of hepatocytes without excessive alcohol consumption [8]. Failure to diagnose NAFLD early can result in steatohepatitis, cirrhosis, and hepatocellular carcinoma [9]. Common pathophysiological mechanisms are involved in the clinical presentation of NAFLD and IBS [10]. Both conditions are associated with microbial dysbiosis, where an imbalance in the gut microbiota composition contributes to disease progression. This event can lead to an impaired intestinal barrier, resulting in increased gut permeability, known as leaky gut, and subsequent systemic inflammation [11]. In this context, several studies have shown the prevalence of IBS in NAFLD patients to be around 29%, with a 13% increased risk associated with female gender. However, the prevalence of IBS rises significantly with the severity of NAFLD: 11.3% in mild cases, 27.7% in moderate cases, and 58.3% in severe cases [12,13,14]. Furthermore, NAFLD is often related to dysmetabolic features such as type 2 diabetes mellitus (T2DM), hypertension, obesity, and dyslipidemia, which complicates patient management by requiring a multi-disciplinary and highly specialized approach [15]. Hence, in recent years, the terminology NAFLD has been substituted with metabolic dysfunction-associated fatty liver disease (MAFLD) and later with metabolic dysfunction-associated steatotic liver disease (MASLD) [16,17]. The literature reports that IBD is a risk factor for NAFLD development due to a yet poorly elucidated interplay that involves lifestyle, genetic, and epigenetic factors as well as gut microbiota dysbiosis [18,19,20,21]. NAFLD prevalence in patients with IBD is quite heterogeneous due to the different diagnostic techniques used, ranging from 20 to 30% of cases detected by liver ultrasound (US) to around 70% with transient elastography [22,23]. In this regard, its prevalence is higher than in the general population (25%) [24]. Recently, we evaluated the prevalence of US and the clinical features of IBD patients with NAFLD and MAFLD [25,26]. However, there is a lack of observational studies about the prevalence of MASLD in patients with IBD in a real-life setting. Indeed, the application of the new MASLD nomenclature requires further investigation, as, according to recent surveys of a randomly selected cohort of 1016 patients, the prevalence of liver steatosis was 27% (261/1016). However, 247/261 patients (89.2%) overlapped with the new definitions for fatty liver disease. In contrast, the population represented exclusively by the presence of fatty liver and not falling under any of the new definitions was represented by only 2.2% (6/277) of the investigated subjects [27]. This suggests that the new definition of MASLD is more inclusive, capturing that portion of patients with fatty liver who carry cardiometabolic comorbidities [28]. While considering both issues, we focused on the fatty liver condition, mainly evaluating the application of the new MASLD nomenclature to an at-risk and still-not-fully studied population such as that of IBD patients. By doing this, it will be possible to assess other potential causes of fatty liver disease in the IBD population besides metabolic risk. For this reason, the main aim of this cross-sectional study is to assess the US prevalence and the clinical features of MASLD in patients with IBDs.

## 2. Materials and Methods

### 2.1. Patients

We conducted a retrospective study involving Italian patients of both genera attending Renato Dulbecco Teaching Hospital of Catanzaro (Italy) in a period between 1 January 2021 and 31 December 2023, diagnosed with IBD in accordance with international guidelines [29]. To be included, participants had to be (i) aged 18 years or older, and (ii) have undergone a liver US at the time of hospital admission. Exclusion criteria included the following: (i) a history of alcohol or drug abuse, (ii) current or past viral hepatitis infection, (iii) a diagnosis of autoimmune liver diseases, (iv) liver cirrhosis, (v) any cancer diagnosis, and (vi) pregnancy or breastfeeding. Data collected from each patient included the following: (i) demographic and anthropometric information, (ii) details about their IBD condition, (iii) disease phenotype and location, (iv) metabolic features, (v) laboratory test results, and (vi) current medications. Among the 3325 patients screened, 272 had IBD and, for this reason, were enrolled in the study.

### 2.2. Diagnosis of NAFLD and MASLD

All patients underwent liver assessment using US, adhering to the protocols from our previous investigations [25,26]. The US was performed by an experienced operator using the LOGIQ S8 XDclear 2.0+ (GE HealthCare, Milan, Italy) with a 3.5 MHz convex transducer for B-mode imaging. Participants fasted for at least 4 h prior to the exam and followed a low-fiber diet while taking 80 mg of simethicone three times daily for three days before the procedure. Liver steatosis was graded as mild (S1), moderate (S2), or severe (S3). S1 was identified by a slight increase in liver echogenicity and minimal contrast between liver and kidney echoes, S2 exhibited higher liver echogenicity, reduced portal vein wall echoes, noticeable posterior beam attenuation, and greater contrast between liver and kidney echoes, while S3 involved marked beam penetration reduction, the absence of echoes from much of the portal vein wall, and a substantial difference in hepatic and renal echoes. A liver steatosis grade of S1 or higher was used for diagnosis [30]. For NAFLD evaluation, alcohol consumption was defined as less than 210 g per week for men and 140 g per week for women (equivalent to 30 g/day for men and 20 g/day for women) [31]. Diagnostic criteria for MASLD were applied based on Rinella et al.‘s guidelines, requiring the presence of liver steatosis alongside at least one of five cardiometabolic risk factors: (i) body mass index (BMI) ≥ 25 kg/m^2^ or waist circumference >94 cm for men and >80 cm for women; (ii) fasting blood glucose ≥ 100 mg/dL or a diagnosis and/or treatment for T2DM; (iii) blood pressure ≥ 135/85 mmHg or the use of antihypertensive medications; (iv) triglycerides ≥ 150 mg/dL or use of lipid-lowering medication; and (v) high-density lipoproteins (HDL) ≤ 40 mg/dL in men and ≤50 mg/dL in women, or treatment with lipid-lowering drugs [17]. Information on past medical history, laboratory results, and endoscopic findings was gathered, and if laboratory and endoscopy data were missing, results from tests performed within 15 days before or after the liver US were used.

### 2.3. Statistical Analysis

We presented numerical variables as mean ± standard deviation (SD) and nominal variables as absolute counts and percentages. Continuous variables were compared using the Mann–Whitney U test after confirming normality with the Shapiro–Wilk test. Categorical variables were analyzed using the chi-square (χ^2^) test. Bivariate logistic regression was performed to identify independent variables. Statistical significance was determined at a *p*-value < 0.05. Data analysis was conducted using SPSS 26.0 software (IBM Corp., Armonk, NY, USA).

## 3. Results

### 3.1. Characteristics of Participants

Table 1 outlines the principal clinical and laboratory features of the individuals included in our investigation. Most IBD patients enrolled were females (*n* = 218, 80%) with a BMI of 24 ± 4 Kg/m^2^ and a waist circumference of 91 ± 12 cm. Only *n* = 19 (7%) were active smokers. Most of these had UC (*n* = 178, 65%), with pancolitis (*n* = 91; 51%) and a full Mayo Score of 2 ± 1. Disease duration was 13 ± 11 years, and the age at onset was 26 ± 15 years, while *n* = 68 (25%) and *n* = 29 (11%) of IBD patients showed an active disease and extraintestinal manifestations, respectively. As for dysmetabolic comorbidities, *n* = 17 (6%), *n* = 41 (15%), and *n* = 21 (7%) of enrolled patients showed T2DM, hypertension, and dyslipidemia, respectively. Most IBD patients were treated with salicylates (*n* = 129, 47%) and biological therapy (*n* = 122, 45%). Surgical treatment was reported in *n* = 49 of patients (18%).

### 3.2. NAFLD and MASLD Prevalence in Patients with IBD

The US prevalence of NAFLD and MASLD among individuals with IBD was 6% and 18%, respectively.

### 3.3. Comparison and Bivariate Logistic Regression Analysis between Study Cohorts

As outlined in Table 2, participants with IBD were categorized based on the presence of NAFLD and MASLD, respectively.

When comparing IBD-MASLD patients to those with IBD-NAFLD, all male patients in the IBD-NAFLD group [*n* = 18 (100%) vs. *n* = 34 (71%), *p* = 0.006] were significantly younger (43 ± 15 vs. 53 ± 13 years, *p* = 0.020) and had lower BMI (23 ± 1 vs. 27 ± 5 kg/m^2^, *p* < 0.001) and waist circumference (86 ± 1 vs. 101 ± 11 cm, *p* < 0.001). None of the IBD-NAFLD patients were active smokers [*n* = 0 vs. *n* = 2 (4%), *p* = 0.526]. In contrast, a significant proportion of IBD-MASLD patients had T2DM [*n* = 9 (19%) vs. 0, *p* = 0.045] and hypertension [*n* = 18 (37%) vs. 0, *p* = 0.012], while there was no significant difference in dyslipidemia between the two groups [*n* = 5 (10%) vs. *n* = 1 (9%), *p* = 0.622]. In terms of laboratory results, IBD-MASLD patients showed higher triglyceride levels (121 ± 55 vs. 81 ± 17 mg/dL, *p* = 0.001), fasting blood glucose (94 ± 23 vs. 83 ± 8 mg/dL, *p* = 0.019), fasting insulin (12 ± 9 vs. 7 ± 2 mg/dL, *p* = 0.001), and homeostasis model assessment of insulin resistance (HOMA-IR; 3 ± 2 vs. 1.5 ± 0.45 mg/dL, *p* = 0.002). They also had lower HDL cholesterol levels (49 ± 15 vs. 56 ± 8 mg/dL, *p* = 0.031). Other laboratory results showed no significant differences between the two groups. Most IBD-NAFLD patients had UC [*n* = 12 (67%) vs. *n* = 30 (62.5%), *p* = 0.495], but CD showed a significantly more frequent ileal involvement [*n* = 6 (100%) vs. *n* = 8 (45%), *p* = 0.027] and an inflammatory disease phenotype [*n* = 6 (100%) vs. *n* = 5 (28%), *p* = 0.005]. IBD-MASLD patients had higher disease activity scores, including the Harvey–Bradshaw Index (5 ± 2 vs. 3 ± 3, *p* = 0.003) and Full Mayo Score (2.3 ± 1 vs. 1.7 ± 1, *p* = 0.003). Additionally, more IBD-MASLD patients required surgery [*n* = 13 (27%), *p* = 0.009]. Regarding treatment, most IBD-MASLD patients were treated with salicylates [*n* = 23 (48%) vs. *n* = 8 (44%), *p* = 0.511] and biological therapies [*n* = 24 (50%) vs. *n* = 8 (44%), *p* = 0.511], with no significant differences between groups. Lastly, bivariate logistic regression revealed that elevated triglyceride levels were significantly associated with IBD-MASLD after adjusting for age (odds ratio [OR] = 1.033, 95% confidence interval [CI] 1.007–1.059, *p* = 0.012).

## 4. Discussion

Liver steatosis is a frequent hepatobiliary manifestation in patients with IBD [32]. Fatty deposition in the hepatocytes and the possible subsequent inflammation can be directly related to the severity of the IBDs as well as to nutritional status and corticosteroid use [33]. Considering the recent change in the nomenclature definition of fatty liver disease from NAFLD to MASLD, we have studied their relationship and a possible pathogenetic way with IBDs. In this context, our analysis shows a US prevalence of NAFLD and MASLD among IBD patients of 6% and 18%, respectively. On the one hand, these findings are in line with our preliminary results, reporting a US prevalence of fatty liver disease associated with dysmetabolic comorbidities of 23% in another cohort of IBD patients [25,26]. On the other hand, we were able to identify a cohort of IBD patients who have fatty liver disease that is not related to cardiometabolic comorbidities and, therefore, does not fall under the new MASLD nomenclature. The prevalence of 6% shown in our IBD-NAFLD cohort is three times more than the prevalence of 2.2% shown by Song et al., although the survey was performed in the general population [27]. This finding confirms that IBDs are a risk factor for fatty liver disease, allowing us to hypothesize how localized inflammatory events in the gut–liver axis may be a prompter for the establishment of liver steatosis. Accordingly, IBD-NAFLD patients show gut dysbiosis that could support this evidence [34]. Currently, the only epidemiological data on this association reported a US prevalence of MASLD among IBD-lean patients of 21% [35]. At the same time, MASLD is an independent predictor of extra-hepatic comorbidities in IBD patients [36]. However, more investigations are needed to better define the exact prevalence of fatty liver disease in IBDs. Indeed, few studies have been performed in the general population, with an extensive range of results (among 10–84%) due to the variability of the diagnostic tools used and the different characteristics of selected patients [27,37,38,39,40]. When compared to the IBD-NAFLD group, most of the patients with IBD-MASLD included in our analysis were males who were older and had a later onset. Recent epidemiological studies demonstrated how IBD homogeneously affects women and men between the second and third decades of life [41,42]. Indeed, when considering the aggregated data of all our IBD patients, the female gender is more prevalent than the male gender (80% vs. 20%). However, liver steatosis and the associated cardiometabolic comorbidities are more frequent in males after the fifth decade of life [43]. This evidence confirms our previous data, in which the IBD-NAFLD group is exclusively represented by males, while the IBD-MASLD group shows a higher prevalence of males than females (71% vs. 29%). Patients with CD and MASLD showed significantly higher disease index based on the Harvey–Bradshaw Index than patients with CD and NAFLD. Likewise, patients with UC and MASLD had a significantly higher disease index, considering the Full Mayo Score, than patients with UC and NAFLD [29]. These results may be related to the mutual relationship between disease activity, extent of disease, and cardiometabolic comorbidities [21]. For this reason, a significantly higher percentage of IBD-MASLD patients were undergoing surgery [44]. As reported in Table 2, our IBD-MASLD patients had significantly higher levels of anthropometric parameters than the IBD-NAFLD group, especially BMI and waist circumference. On the contrary, in the study by Martínez-Domínguez et al., IBD was an independent risk factor related to MASLD in lean patients [35]. The new MASLD nomenclature requires lower waist circumference values to be diagnosed than MAFLD (94/80 cm for the diagnosis of MASLD vs. 102/88 cm for the diagnosis of MAFLD) [16,17]. In a recent study, it was noted that the distinction between MAFLD and MASLD primarily lies in the categorization of lean individuals. While MASLD requires patients to exhibit one metabolic risk abnormality, MAFLD necessitates two. This discrepancy can result in overdiagnosis or misclassification of individuals who do not possess a high metabolic risk as per the MASLD criteria [45]. Moreover, besides higher BMI and waist circumference levels, our IBD-MASLD patients showed changes in glucose and lipid profile and, in particular, significantly higher levels of triglycerides, fasting blood glucose, fasting insulinemia, HOMA-IR, and significantly lower levels of HDL than IBD-NAFLD group, according to recent studies [37,39]. However, although insulin resistance is often associated with several inflammatory conditions, this parameter is not always detectable for IBD patients, especially those with less disease activity. Indeed, Carrillo-Palau et al. highlighted that NAFLD is related to the dysfunction of the beta cells in patients affected by IBD, promoting the development of insulin resistance [46]. Furthermore, our IBD-MASLD group showed a significantly higher percentage of cardiometabolic comorbidities, while De A et al. reported a lower prevalence of T2DM and hypertension (15% and 11%, respectively) in their MASLD-lean patients [40]. According to He et al., the updated nomenclature provides a more precise identification of individuals at higher risk for T2DM [38]. Finally, triglyceride levels were significantly associated with the presence of IBD-MASLD after bivariate logistic regression. Similar data are reported in the literature, but in patients with NAFLD and incident NAFLD after a four-year follow-up [47,48]. The primary limitations of our study include its small sample size, retrospective design, and the absence of a control group of healthy individuals. At the same time, the decision not to include IBD alone as a control group is motivated by the intention to compare two different nomenclatures in an at-risk population such as that of IBD patients. Furthermore, as a cross-sectional study, we cannot identify an etiologic hypothesis attributable to cardiometabolic factors. Finally, the possibility of a genetic predisposition to fatty liver disease, other than transient elastography, could not be assessed. The new MASLD nomenclature has been proposed by the Delphi consensus statement signed by 236 experts on steatotic liver disease from 56 countries and supported by national and international hepatological societies. It includes the presence of cardiometabolic comorbidities, thus increasing the knowledge of the pathophysiological mechanisms concomitant to hepatic steatosis and promoting a multidisciplinary approach to patient management [17,49]. Indeed, patients with both fatty liver disease and IBD have a worse prognosis in terms of mortality than patients with IBD alone [50]. However, there are still few studies in the literature addressing this issue because it was only recently proposed by the scientific community. For this reason, applying this new nomenclature to fatty liver disease in the clinical setting of IBDs can improve their management.

## 5. Conclusions

Our study shows how patients with liver steatosis have cardiometabolic comorbidities ascribable to the new definition of MASLD. In addition, the high prevalence of MASLD in the clinical setting of IBDs indicates a pivotal role in the early diagnosis of hepatic fat accumulation in patients with IBDs, and therefore at risk. At the same time, it is necessary to identify new potential biomarkers as predictors of MASLD risk in IBD patients. New studies in larger population cohorts are needed to confirm our data and evaluate the role of IBDs as a risk factor for MASLD onset and progression.

## Figures and Tables

**Table 1:** Clinical and laboratory features of enrolled IBD patients.

	IBD (N = 272)
Demographic and anthropometric	
Age (years)	46 ± 16
Male gender, *n* (%)	54 (20)
Female gender, *n* (%)	218 (80)
Active smokers, *n* (%)	19 (7)
BMI (Kg/m^2^)	24 ± 4
Waist circumference (cm)	91 ± 12
Disease characteristics	
Disease duration (years)	13 ± 11
Age at onset (years)	26 ± 15
Crohn’s Disease, *n* (%)	94 (35)
CD (Harvey Bradshaw index)	6 ± 3
Ulcerative Colitis, *n* (%)	178 (65)
UC (full Mayo Score)	2 ± 1
Active disease, *n* (%)	68 (25)
Extraintestinal manifestations, *n* (%)	29 (11)
Surgery, *n* (%)	49 (18)
CD disease location and phenotype	
Ileal, *n* (%) *	46 (49)
Colonic, *n* (%) *	14 (15)
Ileo-Colonic, *n* (%) *	33 (35)
Upper GI, *n* (%) *	1 (1)
Inflammatory, *n* (%) *	37 (39)
Fistulizing, *n* (%) *	26 (28)
Stenosing, *n* (%) *	31 (33)
UC disease location	
Proctitis, *n* (%) *	10 (6)
Proctosigmoiditis, *n* (%) *	40 (22)
Left-side, *n* (%) *	37 (21)
Pancolitis, *n* (%) *	91 (51)
Dysmetabolic comorbidities	
T2DM, *n* (%)	17 (6)
Hypertension, *n* (%)	41 (15)
Dyslipidemia, *n* (%)	21 (7)
Laboratory parameters (mean ± SD)	
ALT (UI/L)	19 ± 10
AST (UI/L)	19 ± 8
Total cholesterol (mg/dL)	168 ± 39
LDL (mg/dL)	102 ± 33
HDL (mg/dL)	56 ± 15
Triglycerides (mg/dL)	98 ± 45
Fasting blood glucose (mg/dL)	87 ± 17
Fasting insulinemia (mg/dL)	9 ± 6
HOMA-IR	2 ± 2
CRP (mg/L)	6 ± 10
Fecal calprotectin (mcg/gr)	591 ± 1296
Medications	
Salicylates, *n* (%)	129 (47)
Azathioprine, *n* (%)	40 (15)
>3 cycles of steroids, *n* (%)	20 (7)
Biological therapy, *n* (%)	122 (45)
Anti-TNF-α, *n* (%)	81 (30)
Vedolizumab, *n* (%)	27 (10)
Ustekinumab, *n* (%)	15 (5)
>1 Biological drug, *n* (%)	26 (9)
Current biological therapy duration (years)	3 ± 2
Total biological therapy duration (years)	4 ± 4

Abbreviations: IBD, inflammatory bowel disease; BMI, body mass index; CD, Crohn’s disease; UC, ulcerative colitis; T2DM, type 2 diabetes mellitus; ALT, alanine aminotransferase; AST, aspartate aminotransferase; LDL, low-density lipoprotein; HDL, high-density lipoprotein; HOMA-IR, homeostasis model assessment of insulin resistance; CRP, C-reactive protein; TNF-α, tumor necrosis factor-alfa. * *n*, (%) was evaluated with regard to CD and UC patients, respectively.

**Table 2:** Comparison of IBD patients according to NAFLD and MASLD diagnosis.

	IBD-NAFLD (N = 18)	IBD-MASLD (N = 48)	*p*-Value
Demographic and anthropometric			
Age (years)	43 ± 15	53 ± 13	0.020
Male gender, *n* (%)	18 (100)	34 (71)	0.006
Female gender, *n* (%)	0	14 (29)	
Active smokers, *n* (%)	0	2 (4)	0.526
BMI (Kg/m^2^)	23 ± 1	27 ± 5	<0.001
Waist circumference (cm)	86 ± 1	101 ± 11	<0.001
Disease characteristics			
Disease duration (years)	17 ± 11	15 ± 11	0.267
Age at onset (years)	26 ± 15	39 ± 15	0.004
Crohn’s Disease, *n* (%)	6 (33)	18 (37.5)	0.495
CD (Harvey Bradshaw index)	3 ± 3	5 ± 2	0.003
Ulcerative Colitis, *n* (%)	12 (67)	30 (62.5)	0.495
UC (full Mayo Score)	1.7 ± 1	2.3 ± 1	0.003
Active disease, *n* (%)	4 (22)	11 (23)	0.616
Extraintestinal manifestations, *n* (%)	2 (11)	7 (15)	0.533
Mild steatosis, *n* (%)	10 (55)	28 (58)	0.761
Moderate steatosis, *n* (%)	6 (33)	16 (33)	
Severe steatosis, *n* (%)	2 (12)	4 (9)	
Surgery, *n* (%)	0	13 (27)	0.009
CD disease location and phenotype			
Ileal, *n* (%) *	6 (100)	8 (45)	0.027
Colonic, *n* (%) *	0	3 (17)	
Ileo-Colonic, *n* (%) *	0	6 (33)	
Upper GI, *n* (%) *	0	1 (5)	
Inflammatory, *n* (%) *	6 (100)	5 (28)	0.005
Fistulizing, *n* (%) *	0	4 (22)	
Stenosing, *n* (%) *	0	9 (50)	
UC disease location			
Proctitis, *n* (%) *	1 (8)	2 (7)	0.770
Proctosigmoiditis, *n* (%) *	2 (17)	7 (23)	
Left-side, *n* (%) *	4 (33)	7 (23)	
Pancolitis, *n* (%) *	5 (42)	14 (47)	
Dysmetabolic comorbidities			
T2DM, *n* (%)	0	9 (19)	0.045
Hypertension, *n* (%)	0	18 (37)	0.012
Dyslipidemia, *n* (%)	1 (9)	5 (10)	0.622
Laboratory parameters (mean ± SD)			
ALT (UI/L)	24 ± 7	23 ± 12	0.395
AST (UI/L)	25 ± 9	22 ± 9	0.170
Total cholesterol (mg/dL)	175 ± 17	169 ± 45	0.255
LDL (mg/dL)	106 ± 21	105 ± 37	0.594
HDL (mg/dL)	56 ± 8	49 ± 15	0.031
Triglycerides (mg/dL)	81 ± 17	121 ± 55	0.001
Fasting blood glucose (mg/dL)	83 ± 8	94 ± 23	0.019
Fasting insulinemia (mg/dL)	7 ± 2	12 ± 9	0.001
HOMA-IR	1.5 ± 0.45	3 ± 2	0.002
CRP (mg/L)	5 ± 3	7 ± 13	0.324
Fecal calprotectin (mcg/gr)	215 ± 378	464 ± 929	0.594
Medications			
Salicylates, *n* (%)	8 (44)	23 (48)	0.511
Azathioprine, *n* (%)	2 (11)	3 (6)	0.416
>3 cycles of steroids, *n* (%)	2 (11)	3 (6)	0.416
Biological therapy, *n* (%)	8 (44)	24 (50)	0.511
Anti-TNF-α, *n* (%)	6 (33)	16 (32)	0.610
Vedolizumab, *n* (%)	2 (11)	3 (6)	0.367
Ustekinumab, *n* (%)	0	5 (11)	0.192
>1 Biological drug, *n* (%)	0	6 (12)	0.135
Current biological therapy duration (years)	4 ± 3	3 ± 3	0.724
Total biological therapy duration (years)	6 ± 3	5 ± 3	0.287

Abbreviations: IBD, inflammatory bowel disease; BMI, body mass index; CD, Crohn’s disease; UC, ulcerative colitis; T2DM, type 2 diabetes mellitus; ALT, alanine aminotransferase; AST, aspartate aminotransferase; LDL, low-density lipoprotein; HDL, high-density lipoprotein; HOMA-IR, homeostasis model assessment of insulin resistance; CRP, C-reactive protein; TNF-α, tumor necrosis factor-alfa. * The *p*-value was evaluated with regard to CD and UC patients, respectively.

## Data Availability

The original contributions presented in the study are included in the article, further inquiries can be directed to the corresponding author.

## Abbreviations

ALT	alanine aminotransferase
AST	aspartate aminotransferase
BMI	body mass index
CD	Crohn’s disease
CI	confidence interval
CRP	C-reactive protein
HDL	high-density lipoprotein
HOMA-IR	homeostasis model assessment of insulin resistance
IBD	inflammatory bowel disease
IBS	irritable bowel syndrome
LDL	low-density lipoprotein
MAFLD	metabolic dysfunction-associated fatty liver disease
MASLD	metabolic dysfunction-associated steatotic liver disease
NAFLD	non-alcoholic fatty liver disease
OR	odds ratio
S1	mild liver steatosis
S2	moderate liver steatosis
S3	severe liver steatosis
SD	standard deviation
T2DM	type 2 diabetes mellitus
TNF-α	tumor necrosis factor-alfa
UC	ulcerative colitis
US	ultrasound
χ^2^	chi-square
